# Opening communication channels with people living with HIV using mobile phone text messaging: insights from the CAMPS trial

**DOI:** 10.1186/1756-0500-6-131

**Published:** 2013-04-04

**Authors:** Lawrence Mbuagbaw, Lehana Thabane, Pierre Ongolo-Zogo

**Affiliations:** 1 Centre for Development of Best Practices in Health (CDBPH), Yaoundé Central Hospital, Avenue Henri Dunant, Messa, PO Box 87, Yaoundé, Cameroon; 2 Department of Clinical Epidemiology and Biostatistics, McMaster University, Hamilton, ON, Canada; 3 Biostatistics Unit, Father Sean O’Sullivan Research Centre, St Joseph’s Healthcare, Hamilton, ON, Canada

**Keywords:** Text messaging, SMS, HIV, Adherence, Antiretroviral therapy, Communication, Cameroon, CAMPS trial

## Abstract

**Background:**

Using two-way mobile phone text messages to improve adherence to antiretroviral medication enhances communication between patients and health workers. We describe the implications of participants’ responses to text messages in the Cameroon Mobile Phone SMS (CAMPS) trial.

**Findings:**

This is a cross-sectional analysis of data from the intervention arm of the CAMPS trial. CAMPS was a randomized controlled trial of motivational text messaging versus usual care to improve adherence to antiretroviral medication among people living with HIV in Yaounde, Cameroon (n = 200) over a 6 month period. Participants in the intervention arm (n = 101) were given a contact phone number, but were not required to respond to their reminder messages. If they did, their responses were noted and reported as counts and percentages. We received 99 phone calls and 55 text messages (154 responses) from 48 participants during the study period. The median number of responses was 1 (first quartile [Q1]: 1; third quartile [Q3]: 3). Half (n = 79, 51.1%) of them were expressions of gratitude. The rest included requests for logistical (n = 21, 13.6%), medical (n = 20, 12.9%) and financial (n = 11, 7.1%) support.

**Conclusion:**

Initiating two-way mobile communication opens more channels for people living with HIV to express unmet needs. Researchers, policy makers and clinicians should be ready to respond to the needs expressed by patients who respond to text messages.

**Trial registration:**

Pan-African Clinical Trials Registry: PACTR201011000261458;

Clinicaltrials.gov: NCT01247181

## Findings

### Health communication using mobile phones

Despite numerous advancements in Human Immunodeficiency Virus (HIV) care and treatment, poor adherence is still a potential threat to universal access to antiretroviral therapy (ART) [1]. Due to the multifactorial nature of the determinants of patient adherence [2] and diversified research efforts to respond to each of these factors, mobile phones have emerged as a potentially useful tool to improve adherence rates [3]. Due to their ubiquity in countries most affected by HIV [4], the use of mobile phones to improve HIV related health outcomes is receiving more attention as emerging evidence suggests reminder messages can increase adherence to ART and retention in care, decrease viral load and treatment interruptions, and improve communication with healthcare personnel [5-7]. Such mobile health (mHealth) interventions can also be used to address other health behaviors and to provide useful and timely information to health workers [8]. Other studies report the potential of using the SMS for other types of health related communication and the promotion of additional health services, yet there is limited information as to how this can be achieved [5,6].

The relative novelty of text messaging as part of mHealth results in a lack of generalizable policy frameworks and guidelines for care. Health text messaging guidelines developed by the United States Department for Human and Health Services do not cover how to manage direct feedback and recommend using text messaging as part of a larger communication strategy [9]. For such frameworks and guidelines to be implemented, the issues most relevant to patients or the success of mHealth must be taken into account.

Although automated mobile phone text messages are less labor intensive than manually composed messages, they may not permit feedback to be addressed in real time and thus neglect urgent concerns from the responders. Likewise, standardized messages may contain insufficient or unclear information and prompt the need to address other related issues. Furthermore, urgent responses may be missed if they are sent after working hours when mHealth phones are not monitored. Given the above, the expectations for the parties on the sending end and the responsibilities of those on the receiving end need to be investigated. As such, we pose the following question: *what do people living with HIV hope to benefit from text communication with health workers and how can health workers prepare to respond to the needs identified through text messaging?*

The responses and feedback of clients who receive text messages can provide important clues as to the most effective way of designing and organizing text messaging interventions. The aim of this paper is to describe the responses from the participants in the Cameroon Mobile Phone SMS (CAMPS) trial, [10,11] and the implications for health service providers.

## Methods

**Figure 1:**
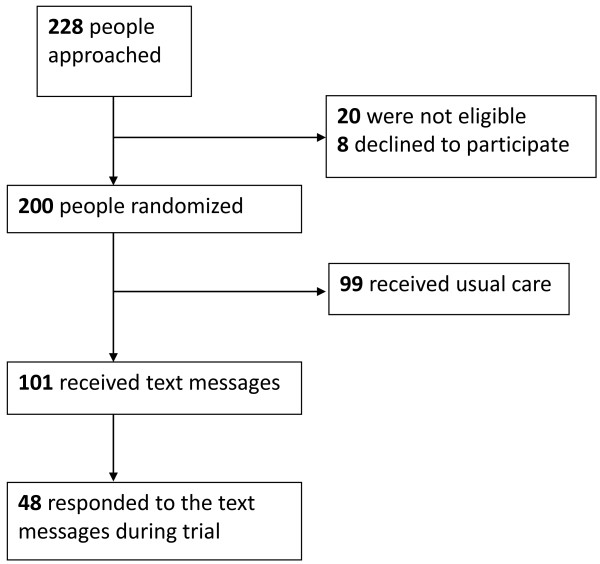
Flow diagram of study.

We conducted a cross-sectional analysis of the responses from participants in the intervention arm of the CAMPS trial. The CAMPS trial was a two-arm single blinded randomized controlled trial of weekly motivational mobile phone text messages versus usual care for improving adherence to ART, at the Yaoundé Central Hospital in Cameroon. Ethical clearance was obtained from the Cameroon National Ethics Committee (authorization number 172/CNE/SE/2010). All participants included in the study provided verbal and written consent. The methods are described in detail elsewhere [10,11]. In brief, 200 eligible participants were randomized to receive text messages (n = 101) or usual care (n = 99). Usual care involved adherence counseling as determined by care givers. Eligibility was determined based on age (21 or older), ownership of a mobile phone, use of ART for at least one month, and willingness to participate in the trial. The trial ran from November 2010 to July 2012 (Figure 1). The motivational text messages were sent once a week and included a phone number that participants could call if they required assistance. Participants did not receive communication credit so all the responses occurred at their expense. We noted the content of the responses (phone calls and text messages) and grouped them into categories: expressions of gratitude, inquiries about the trial, logistical support (related to provision of services), medical, counseling, requests for financial assistance, and others. Participants who left “missed calls” were called back; this was noted as a phone call. The phone number provided to the participants in the text message was different from the one from which the messages were sent. Participants who accidentally responded to the sending number were redirected to the “help” number. The help number phone connected to an experienced HIV clinician (available twenty-four hours a day every day) who was part of the research team, but not clinic staff, and therefore did not have access to patient records. Based on the participants’ inquiry they were channeled to the appropriate department of the HIV clinic. The participant characteristics were determined by matching the responses to the phone numbers collected at baseline.

## Results

All messages received could be linked to participants in the intervention arm of the trial (mean age 42.6 years [Standard Deviation - SD 9.80]; 35 [72.9%] were female). All had received some formal education. Most patients had disclosed their HIV status to family members (n = 43, 89.6%) and were on a first line ART regimen (n = 41, 91.1%). The median duration on ART was 33 months (first quartile [Q1]: 17.25; third quartile [Q3]: 56.25). Detailed socio-demographic and clinical characteristics are reported in Table 1 (see Table 1).

**Table 1:** Socio-demographic characteristics of participants who responded to messages in the CAMPS trial (n = 48)

**Variable**	**Statistic**
**Age (years): mean (SD)**	42.6 (9.80)
**Gender: count (%)**	
**Female**	35 (72.9)
**Level of education: count (%)**	
**Primary**	20 (41.7)
**Secondary**	22 (45.8)
**University**	6 (12.5)
**Family aware of HIV status: count (%)**	43 (89.6)^μ^
**CDC**^**¥ **^**classification - AIDS**^**£ **^**defining illness: count (%)**	32 (69.6)^β^
**Regimen: count (%)**	^α^
**First line**	41 (91.1)
**Second line**	4 (8.9)
**Duration on ART (months): median (Q1, Q3)**	33.0 (17.25, 56.25)
**Adherence/Visual Analogue Scale: mean (SD)**	88.15 (14.351)
**Quality of life **^α^**: mean (SD)**	3.2 (0.40)

^μ^1 missing; ^β^2 missing; ^α^3 missing; Quality of life was measured using Short Form −12 (http://www.sf-36.org/tools/sf12.shtml); SD: standard deviation, ^¥^CDC: Centers for disease control and prevention; Q1: first quartile; Q3: third quartile; ^£^AIDS: Acquired Immune Deficiency Syndrome.

Of the 101 participants who received text messages, 48 (47.5%) responded at least once either by phone call or by text message. We received 99 phone calls and 55 text messages (154 responses) over a period of six months. These responses tended to cluster around the day the messages were sent. The median number of responses was 1 (Q1: 1; Q3: 3). Responses came in as early as 7.30 am and as late as 10.30 pm. Characteristics of responses are reported in detail in Table 2 (see Table 2).

**Table 2:** Characteristics of responses to messages in the CAMPS trial

**Content of message**	**Mode of contact**		Count (%)*
	**Phone call**	**Text message**	
**Expression of gratitude**	35	44	79 (51.1)
**Logistical**	19	2	21 (13.6)
**Medical**	19	1	20 (12.9)
**Other**	9	3	12 (7.8)
**Financial assistance**	7	4	11 (7.1)
**Inquiries about study**	8	1	9 (5.8)
**Counseling**	2	0	2 (1.2)
**Total**	**99**	**55**	**154**

*Percentages may not add up to 100 due to rounding off.

Half of them (n = 79, 51.1%) were expressions of gratitude. Some participants (n = 9, 5.8%) wished to verify the authenticity of the text messages and who was responsible for sending them. Respondents who required medical help (n = 20, 12.9%) complained about side-effects and other ailments; how to handle unwanted pregnancies, how to take medication, and what to do after missing a dose (see Table 2).

Messages for logistical support (n = 21, 13.6%) included requests for assistance in collecting results from laboratory, re-scheduling appointments, complaints of stock-outs at the hospital pharmacy, and requests to ameliorate clinic wait times.

Counseling was requested regarding HIV testing within a committed relationship (n = 2, 1.2%). Financial help (n = 1, 7.1%) was sought for a variety of reasons, including setting up small businesses and other personal requests.

The last group (classified as other) included messages from participants reporting that their phones were missing (and providing a new contact number), reporting the death of the owner of the phone, and requesting to withdraw from the study (n = 12, 7.8%).

## Discussion

This observational study within a randomized clinical trial highlights the needs of people with HIV and weaknesses within the health system identified through the lens of new and rapidly spreading mobile phone technology.

First, it must be noted that the use of a text message reminder system was overall appreciated and half of the responders communicated only to express their gratitude. These results coincide with other studies which have documented high levels of satisfaction with two-way text messaging [6,12].

Secondly, these messages highlighted unmet needs regarding disease counseling. Patients contacted the research team seeking additional information regarding medication procedures, consequences of and compensation for a missed dose, and expected side effects.

Third, these findings provide a comment on the human resource shortages and organizational difficulties which plague many health systems in resource limited countries - often demonstrated by long waiting times, stock-outs, and delays in obtaining lab reports [13,14]. The most dangerous of these is the frequent stock-outs of ART. They are a major cause of poor adherence and directly counteract adherence enhancing interventions [13]. They also reduce patients’ confidence in an already fragile health system.

A major barrier to the success of mHealth programs is the unavoidable interaction with other weak components of the health system [14,15]. For example, it is difficult to enhance adherence through text messaging when the medication is unavailable or insufficient human resources prevents timely quality care. The potential for text messaging to enhance care may be limited by these health system challenges and therefore scaling up the program would require additional resources to handle the new demands.

Other potential costs of routine use of text messaging for health care needs must also be considered. Such costs would include the cost of technology and additional staffing. Although additional, the relatively low cost of text messaging and staff willingness to participate in text messaging programs in Cameroon are encouraging factors that may boost the uptake of a cost-effective adherence program [16].

Fourthly, these communications have demonstrated the feasibility of using mobile phone technology as a means to open additional communication channels with patients beyond clinical hours and to provide professional assistance in real time. It is important to note the level of commitment and expertise utilized in the CAMPS trial. The recipient of each response should be well versed in, or able to channel patients to the necessary assistance. In future renditions of this strategy, it would be desirable to have a toll free number, with an experienced HIV clinician, a social worker, or someone capable of locating lab results, to schedule appointments and facilitate the provision of care. Access to patient records may also be desirable in order to provide personalized care. At this point, policy frameworks and guidelines must be developed to assist health workers providing effective, ethical, and efficient remote support [15]. Such guidelines should explicitly state that text messaging does not replace emergency care and should not delay appropriate health care.

This study has some limitations. Of note is the fact that these responses were initiated at the expense of the participants. Even though this may serve as a measure of their need for mobile services, many participants who did not respond to the text messages may not have done so because they lacked communication credit. Therefore, these findings reflect the views of only a portion of the wealthier, more health-conscious participants who received text messages in the CAMPS trial [11].

## Conclusion

Text messaging for adherence to ART is a relatively new intervention. While it is unlikely that large scale messaging can be individually tailored, it is important to identify the needs of those receiving these messages. Researchers, policy makers and clinicians must recognize that two-way text messaging provides space for dialogue for patient needs which can then be addressed. These needs may be logistical, medical or informational.

## Abbreviations

ART: Anti-Retroviral Therapy; CAMPS: Cameroon Mobile Phone SMS trial; CDC: Centres for Disease Control and Prevention; HIV: Human immune deficiency virus; mHealth: Mobile Health; Q1: First quartile; SD: Standard deviation; SMS: Short Message Service.

## Competing interests

The authors declare that they have no competing interests.

## Authors’ contributions

LM and LT designed the study. LM and POZ collected the data. All authors analyzed and interpreted the data; and reviewed all versions of the manuscript. All authors read and approved the final manuscript.

## References

[B1] Gill CJ Hamer DH Simon JL Thea DM Sabin LL No room for complacency about adherence to antiretroviral therapy in sub-Saharan Africa AIDS 2005 19 1243 1249 10.1097/01.aids.0000180094.04652.3b 16052078 PMC6712424

[B2] Osterberg L Blaschke T Adherence to medication N Engl J Med 2005 353 487 497 10.1056/NEJMra050100 16079372

[B3] Lester R Karanja S Mobile phones: exceptional tools for HIV/AIDS, health, and crisis management Lancet Infect Dis 2008 8 738 739 10.1016/S1473-3099(08)70265-2 19022188

[B4] Kaplan WA Can the ubiquitous power of mobile phones be used to improve health outcomes in developing countries? Global Health 2006 2 9 10.1186/1744-8603-2-9 16719925 PMC1524730

[B5] Pop-Eleches C Thirumurthy H Habyarimana JP Zivin JG Goldstein MP de Walque D MacKeen L Haberer J Kimaiyo S Sidle J Mobile phone technologies improve adherence to antiretroviral treatment in a resource-limited setting: a randomized controlled trial of text message reminders AIDS 2011 25 825 834 10.1097/QAD.0b013e32834380c1 21252632 PMC3718389

[B6] Lester RT Ritvo P Mills EJ Kariri A Karanja S Chung MH Jack W Habyarimana J Sadatsafavi M Najafzadeh M Effects of a mobile phone short message service on antiretroviral treatment adherence in Kenya (WelTel Kenya1): a randomised trial Lancet 2010 376 1838 1845 10.1016/S0140-6736(10)61997-6 21071074

[B7] Mukund Bahadur KC Murray PJ Cell phone short messaging service (SMS) for HIV/AIDS in South Africa: a literature review Stud Health Technol Inform 2010 160 530 534 20841743

[B8] Thirumurthy H Lester RT M-health for health behaviour change in resource-limited settings: applications to HIV care and beyond Bull World Health Organ 2012 90 390 392 10.2471/BLT.11.099317 22589574 PMC3341690

[B9] U.S. Department of Health and Human Services (HHS) Text4Health Task Force. Health Text Messaging Recommendations to the Secretaryhttp://www.hhs.gov/open/initiatives/mhealth/recommendations.html

[B10] Mbuagbaw L Thabane L Ongolo-Zogo P Lester RT Mills E Volmink J Yondo D Essi MJ Bonono-Momnougui RC Mba R The cameroon mobile phone sms (CAMPS) trial: a protocol for a randomized controlled trial of mobile phone text messaging versus usual care for improving adherence to highly active anti-retroviral therapy Trials 2011 12 5 10.1186/1745-6215-12-5 21211064 PMC3023676

[B11] Mbuagbaw L Thabane L Ongolo-Zogo P Lester RT Mills EJ Smieja M Dolovich L Kouanfack C The Cameroon Mobile Phone SMS (CAMPS) Trial: A Randomized Trial of Text Messaging versus Usual Care for Adherence to Antiretroviral Therapy PLoS One 2012 7 e46909 10.1371/journal.pone.0046909 23236345 PMC3516507

[B12] Hardy H Kumar V Doros G Farmer E Drainoni ML Rybin D Myung D Jackson J Backman E Stanic A Skolnik PR Randomized controlled trial of a personalized cellular phone reminder system to enhance adherence to antiretroviral therapy AIDS Patient Care STDS 2011 25 153 161 21323532 10.1089/apc.2010.0006 PMC3101947

[B13] Boyer S Clerc I Bonono CR Marcellin F Bile PC Ventelou B Non-adherence to antiretroviral treatment and unplanned treatment interruption among people living with HIV/AIDS in Cameroon: Individual and healthcare supply-related factors Soc Sci Med 2011 72 1383 1392 10.1016/j.socscimed.2011.02.030 21470734

[B14] GIP ESTHER, UNICEF, WHO Evaluation of ARV Procurement and Supply Management Systems in West and Central Africa Region. In Book Evaluation of ARV Procurement and Supply Management Systems in West and Central Africa Region 2008 http://www.unicef.org/wcaro/Wcaro_ARV_PSM_study_En_June_2008.pdf

[B15] Tamrat T Kachnowski S Special Delivery: An Analysis of mHealth in Maternal and Newborn Health Programs and Their Outcomes Around the World Matern Child Health J 2012 16 1092 1101 10.1007/s10995-011-0836-3 21688111

[B16] Mbuagbaw LC Thabane L Ongolo-Zogo P Karanja S Health workers views on the use of text messages to improve adherence to ART: A cross-sectional survey of health workers involved in CAMPS trial Can J Infect Dis Med Microbiol 2012 23 77A

